# Sexual dimorphism in peripheral blood cell characteristics linked to recanalization success of endovascular thrombectomy in acute ischemic stroke

**DOI:** 10.1007/s11239-023-02881-z

**Published:** 2023-08-18

**Authors:** L. Malin Overmars, Wouter W. van Solinge, Hester M. den Ruijter, H. Bart van der Worp, Bram Van Es, Cornelia A. R. Hulsbergen-Veelken, Geert Jan Biessels, Lieza G. Exalto, Saskia Haitjema

**Affiliations:** 1 Central Diagnostic Laboratory, University Medical Center Utrecht, Utrecht University, Utrecht, The Netherlands; 2 Department of Neurology and Neurosurgery, UMC Utrecht Brain Center, University Medical Center, Utrecht University, Utrecht, The Netherlands; 3 Laboratory of Experimental Cardiology, University Medical Center Utrecht, Utrecht University, Utrecht, The Netherlands

**Keywords:** Endovascular thrombectomy, Acute ischemic stroke, Recanalization success, Sex differences, Blood cell characteristics, Stroke etiology

## Abstract

Endovascular thrombectomy (EVT) success to treat acute ischemic stroke varies with factors like stroke etiology and clot composition, which can differ between sexes. We studied if sex-specific blood cell characteristics (BCCs) are related to recanalization success. We analyzed electronic health records of 333 EVT patients from a single intervention center, and extracted 71 BCCs from the Sapphire flow cytometry analyzer. Through Sparse Partial Least Squares Discriminant Analysis, incorporating cross-validation and stability selection, we identified BCCs associated with successful recanalization (TICI 3) in both sexes. Stroke etiology was considered, while controlling for cardiovascular risk factors. Of the patients, successful recanalization was achieved in 51% of women and 49% of men. 21 of the 71 BCCs showed significant differences between sexes  (pFDR-corrected < 0.05). The female-focused recanalization model had lower error rates than both combined [t(192.4) = 5.9, p < 0.001] and male-only models [t(182.6) = − 15.6, p < 0.001]. In women, successful recanalization and cardioembolism were associated with a higher number of reticulocytes, while unsuccessful recanalization and large artery atherosclerosis (LAA) as cause of stroke were associated with a higher mean corpuscular hemoglobin concentration. In men, unsuccessful recanalization and LAA as cause of stroke were associated with a higher coefficient of variance of lymphocyte complexity of the intracellular structure. Sex-specific BCCs related to recanalization success varied and were linked to stroke etiology. This enhanced understanding may facilitate personalized treatment for acute ischemic stroke.

**Supplementary Information:**

The online version contains supplementary material available at 10.1007/s11239-023-02881-z.

## Highlights

- Sex-specific blood cell characteristics (BCCs) vary and are associated with recanalization success in acute ischemic stroke.
- In women, successful recanalization and cardioembolism as the cause of stroke are associated with a higher number of reticulocytes, while unsuccessful recanalization and large-artery atherosclerosis (LAA) as the cause of stroke are associated with a higher mean corpuscular hemoglobin concentration.
- In men, unsuccessful recanalization and LAAas the cause of stroke are associated with a higher coefficient of variance of lymphocyte complexity of the intracellular structure.
- This study highlights the potential of analyzing data from routine care flow cytometry analyzers to identify sex-differences in factors related to acute ischemic stroke, which may ultimately facilitate personalized treatment.

**Supplementary Information:**

The online version contains supplementary material available at 10.1007/s11239-023-02881-z.

## Introduction

The recanalization success of endovascular thrombectomy (EVT) in patients with acute ischemic stroke (AIS) is related to AIS etiology and cellular clot composition [1–4]. Fibrin- and thrombocyte-rich clots, which are more common in cardioembolic stroke [4–6], are associated with unsuccessful recanalization because of greater stiffness and adhesion to the vessel wall [1]. Erythrocyte-rich clots, on the other hand, are more common in atherosclerotic stroke [4, 6], and associated with successful recanalization [4, 5, 7, 8]. AIS etiology is associated with sex, where women are less likely to have atherosclerotic stroke [9, 10].

Other sex differences have been revealed in various pathophysiological processes related to AIS. Women and men have different atherothrombotic plaque phenotypes, and sex differences in gene expression in immune cells following AIS have been noted [11–13]. Additionally, age and sex may interact in the post-stroke inflammatory milieu and influence AIS outcomes [14]. Nonetheless, sex-differences in peripheral blood cell characteristics (BCCs) related to recanalization success of EVT in AIS have not yet been studied but are potentially relevant as these likely reflect clot features. An improved understanding of sexual dimorphism in underlying biological processes of AIS may contribute to more accurate personalized treatment.

In this study, we examined 71 different pretreatment BCCs of patients who underwent EVT at a single stroke intervention center. These BCCs contain numbers, percentages and morphological characteristics of leukocytes, erythrocytes, reticulocytes, and thrombocytes. Several of these characteristics are known to be associated with cardiovascular disease, and are related to atherosclerosis, inflammatory processes, coagulation and anemia, among others [15–20]. Here we test the hypothesis that these readily-obtainable peripheral BCCs associated with recanalization success by EVT in patients with AIS differ by sex, reflecting potential sex-related differences in underlying biological processes of AIS.

## Patients and methods

### Electronic health record extraction

**Fig. 1:**
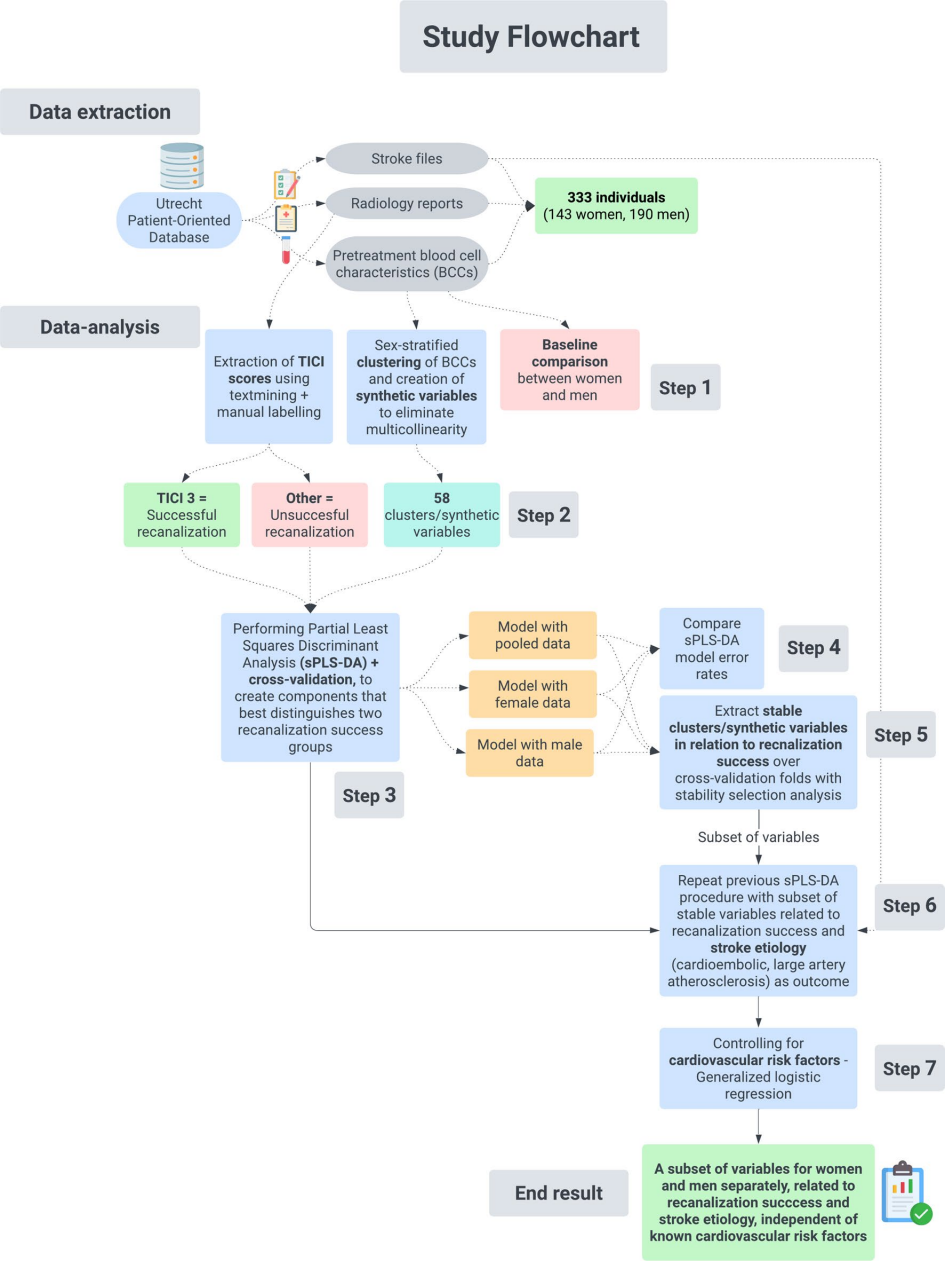
Study flowchart describing the data extraction and analysis proceduress

We selected and analyzed electronic health records (EHRs) from the Utrecht Patient-Oriented Database (UPOD) (Fig. 1). UPOD is an infrastructure of relational databases comprising EHRs for all patients treated at the University Medical Center Utrecht (UMCU). The structure and content of UPOD have been described in more detail elsewhere[21]. UPOD data acquisition and management is following current regulations concerning privacy and ethics. All data were pseudonymized before use in the study. The current study was conducted under the declaration of Helsinki and hospital specific GDPR procedures. The need for informed consent for this study was waived (IRB number 21/038, Medical Research Ethics Committee NedMec).

### Eligibility criteria

We analyzed AIS patients who underwent EVT at UMCU between January 2015 and February 2020 with available BCCs before EVT (Fig. 1). Radiology reports describing the outcome of the EVT procedures were extracted from UPOD. We excluded patients where EVT was not performed due to factors such as hemorrhage, elongated vessels, or thrombus dissolution (20 women (6%); 20 men (5%)), as well as patients without available BCCs within 60 min of arriving at the emergency department (157 women (52%); 138 men (42%)).

### Outcome definition

Recanalization success, the primary outcome, was defined using the TICI scale[22], which was extracted from radiology reports using rule-based text mining (Fig. 1). The TICI scale was chosen as it was relevant to circulating BCCs related to clot type and degree of success in removing the clot. Two categories were defined: successful recanalization (TICI 3) and unsuccessful recanalization (TICI 2a, 2b, 2c, 1, and 0). If no TICI scale or more than one TICI scales were mentioned, the reports were manually labeled by an experienced neurologist (LGE).

### Baseline characteristics

Clinical characteristics of eligible patients, such as cardiovascular risk factors, use of IVT before EVT, and AIS etiology, were derived from stroke files stored in UPOD (Fig. 1). Antiplatelet drugs and direct oral anticoagulant use were also considered due to their potential association with thrombocyte-related BCCs. AIS etiology was classified using the TOAST system [23], and stroke-to-door and door-to-needle times in minutes were extracted from the stroke files.

### Blood cell characteristics

A total of 71 BCCs were extracted using the Sapphire routine flow cytometry hematology analyzer from Abbott Diagnostics. This analyzer used in clinical diagnostics to quantify various characteristics of cells and particles in suspension in a fluid for diagnostic decisions. It uses principles of hydrodynamics, optics, and electronics to accurately measure multiple physical and chemical properties of individual cells or particles as they flow in a narrow stream through a laser beam. The laser beam interacts with the particles, causing them to scatter light and emit fluorescence signals. The analyzer's detectors capture these signals and convert them into structured data that provide insight into cell characteristics such as size, shape, complexity and the presence or absence of specific biomarkers. Time differences between the EVT procedures and Sapphire analyses were calculated, only Sapphire analyses from before EVT were used in the analysis.

The analyses to identify BCCs related to recanalization success and AIS etiology in women and men entailed seven main steps, which are described in detail in Fig. 1 and the Supplementary Methods.

## Results

### Patient characteristics

A total of 333 patients, comprising 143 women (median age = 73.0 years, IQR = 55.5 to 82.0) and 190 men (median age = 71.0 years, IQR = 58.3 to 76.0) were analyzed (Table 1). There were no sex differences in stroke severity (NIHSS). Successful recanalization was achieved in 73 women (51.0%) and 88 men (46.0%), with no significant difference between women and men (χ^2^_Pearson_ = 0.73, p = 0.39). AIS etiology was also similar between women and men (χ^2^_Pearson_ = 1.25, p = 0.74).

**Table 1:** Baseline characteristics of AIS patients who underwent endovascular thrombectomy

	Women (N = 143)			Men (N = 190)		
	N	Successful EVT, N = 73	Unsuccessful EVT, N = 70	N	Successful EVT, N = 88	Unsuccessful EVT, N = 102
Age in years—median (IQR)	143	73.0 (56.0, 83.0)	73.0 (57.5, 83.0)	190	73.0 (66.8, 80.0)	67.0 (57.2, 75.8)
National institutes of health stroke scale (NIHSS) points—median (IQR)	123	12.0 (9.2, 17.0)	13.0 (9.0, 17.0)	172	14.0 (9.0, 18.0)	14.5 (10.0, 17.0)
Intravenous thrombolysis prior to EVT—no. (%)	143	28 (38.4)	19 (27.1)	190	31 (35.2)	36 (35.3)
Cardiovascular risk factors						
Hypertension—no. (%)	137	45 (63.4)	37 (56.1)	185	49 (58.3)	51 (50.5)
Hyperlipidemia—no. (%)	135	27 (38.6)	22 (33.8)	186	41 (48.8)	40 (39.2)
Atrial fibrillation—no. (%)	136	12 (17.1)	9 (13.6)	184	17 (20.2)	17 (17.0)
Diabetes mellitus—no. (%)	136	14 (20.0)	4 (6.1)	186	17 (20.2)	11 (10.8)
Current smoker—no. (%)	107	8 (14.5)	11 (21.2)	148	12 (19.0)	17 (20.0)
History of stroke or transient ischemic attack—no. (%)	136	12 (17.1)	12 (18.2)	185	8 (9.6)	21 (20.6)
History of myocardial infarction—no. (%)	123	3 (4.9)	6 (9.7)	176	18 (23.4)	15 (15.2)
Platelet aggregation inhibitor use—no. (%)	136	16 (22.9)	16 (24.2)	186	29 (34.5)	32 (31.4)
Direct oral anticoagulant use—no. (%)	136	12 (17.1)	7 (10.6)	186	11 (13.1)	11 (10.8)
Treatment times						
Stroke to door in minutes—median (IQR)	44	67.0 (40.5, 89.0)	55.0 (43.0, 100.0)	57	71.0 (50.0, 120.0)	59.5 (38.0, 90.0)
Door to needle in minutes—median (IQR)	46	27.0 (23, 39)	25.0 (19.8, 32.0)	60	25.0 (18.0, 40.0)	29.0 (22.5, 41.0)
Acute ischemic stroke etiology	135			176		
Cardioembolism—no. (%)		34 (46.6)	23 (33.3)		36 (40.9)	28 (28.3)
Large-artery atherosclerosis—no. (%)		19 (26.0)	24 (34.8)		26 (29.5)	36 (36.4)
Undertermined etiology—no. (%)		16 (21.9)	19 (27.5)		25 (28.4)	25 (25.3)

### Sex differences in blood cell characteristics

In total, 71 BCCs were available (Supplemental Table S1). In the blood sample prior to EVT, 21 out of 71 available erythrocyte-, leucocyte-, and thrombocyte-related BCCs were significantly different between women and men (p_FDR-corrected_ < 0.05) (Fig. 1, Step 1; Supplemental Table S2). For example, the platelet count was higher in women (median = 269.0 × 10^9^/L, IQR = 228.0 to 325.3 × 10^9^/L), than in men (median = 217 × 10^9^L, IQR = 178 to 267 × 10^9^/L, p_FDR-corrected_ =  < 0.001) and the hemoglobin level was lower in women (median = 8.5 mmol/L, IQR = 7.9 to 9.1 mmol/L) than in men (median = 9.0 mmol/L, IQR = 8.3 to 9.6 mmol/L) (Supplemental Table S2).

### Clustering of blood cell characteristics

Multicollinearity was present in multiple BCCs (Fig. 1, Step 2; Supplemental Figure S1). Highly collinear BCCs were clustered, based on which synthetic variables representing BCCs clusters were created. The optimal number of clusters was 58 (similar for men and women) (Rand-index 0.98). For example, red blood cell characteristics like hemoglobin and hematocrit were clustered, as well as platelet characteristics like the absolute platelet count by impedance and optics (Supplemental Table S1). The following analyses were based on the 58 BCC clusters (Fig. 1, Step 2). A mapping between the cluster numbers and the corresponding blood cell characteristics can be found in Supplemental Table S1.

### Sex stratification leads to better explanation of recanalization success in women

**Fig. 2:**
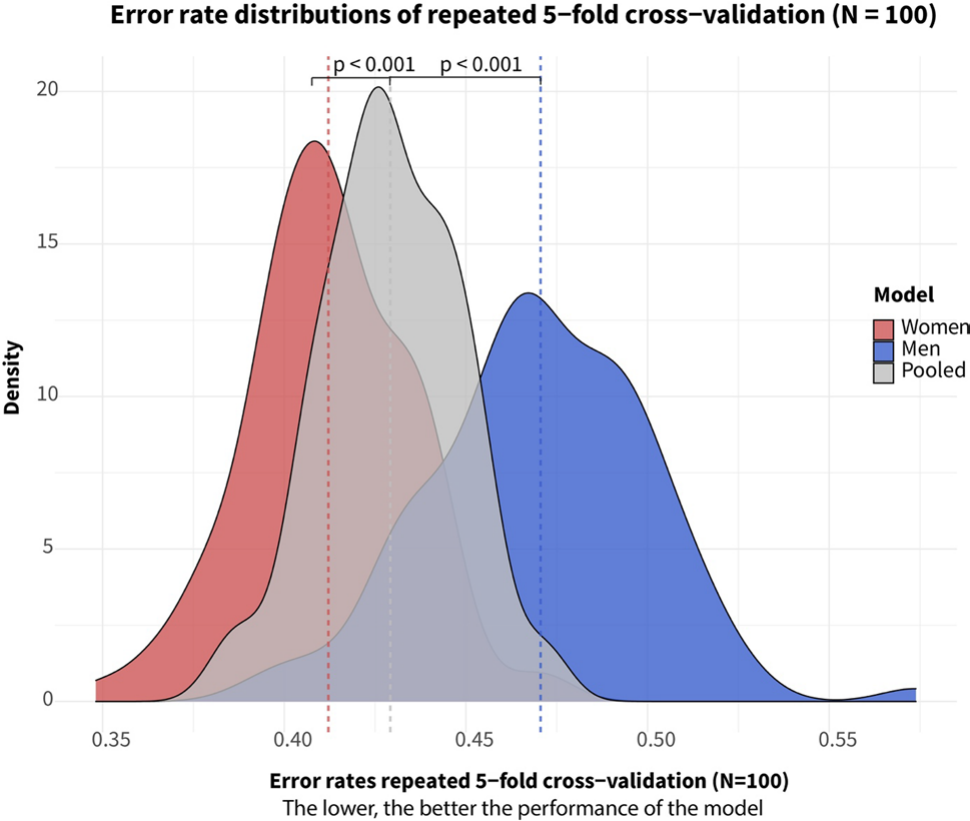
Error rates of the pooled, female, and male sPLS-DA models to distinguish successful from unsuccessful recanalization with blood cell characteristics, based on fivefold cross-validation repeated 100 times. Differences in error rate distributions, defined as the average number of misclassified samples divided by the total number of samples, tested using Welch two sample t-tests

**Fig. 3:**
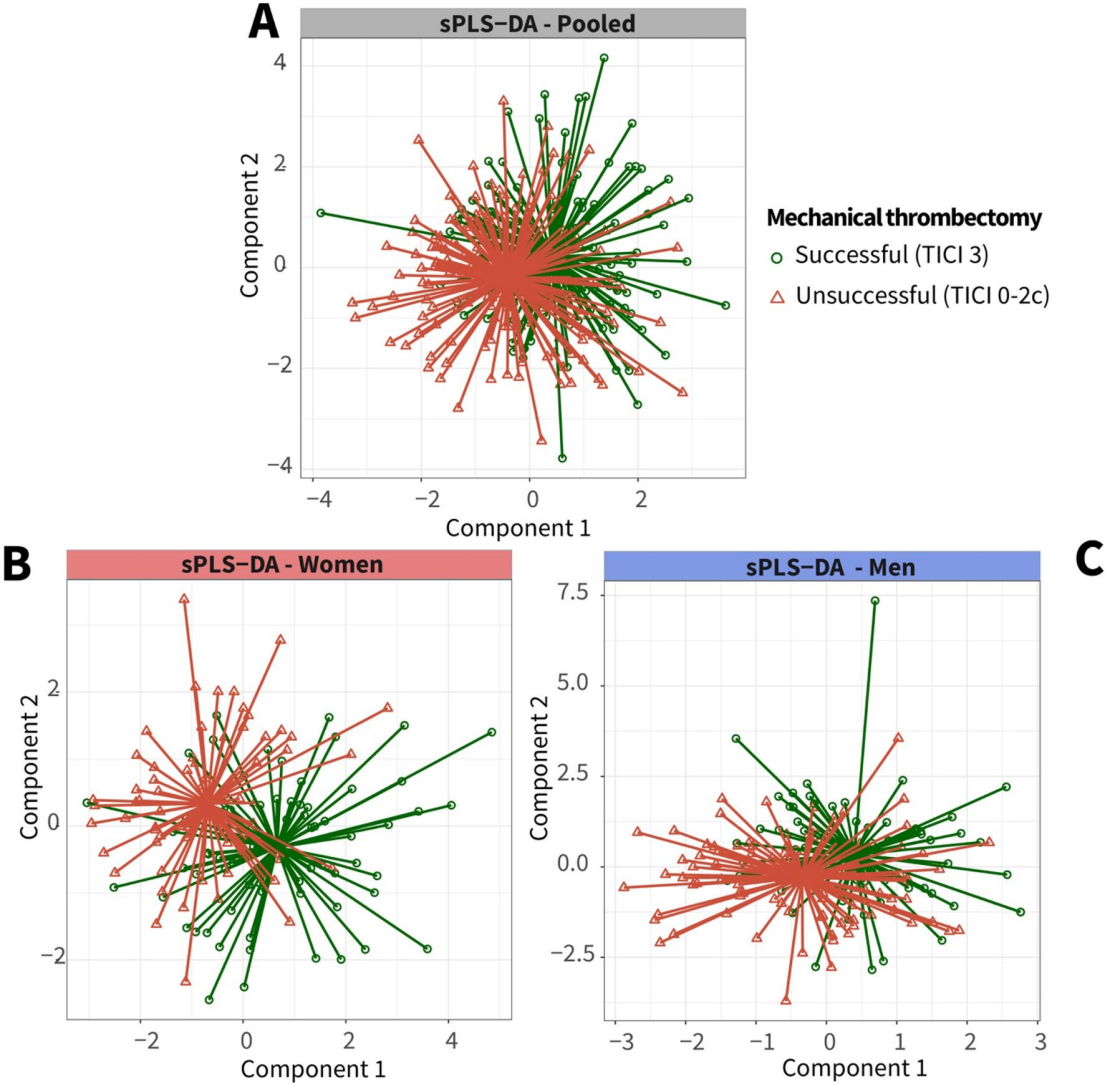
Sparse PLS Discriminant Analysis (sPLS-DA) to distinguish patients with successful (TICI 3) and unsuccessful (TICI 0-2c) recanalization after enodvascular thrombectomy (EVT) based on blood cell characteristics. **A**–**C** Plots of sPLS-DA performed on baseline BCC clusters reflect two components trained to distinguish successful recanalization (green, circles) from unsuccessful recanalization (red, triangles) after endovascular thrombectomy. **A** Results of the sPLS-DA procedure trained on pooled data of women and men; **B** Results of the sPLS-DA procedure trained on data of women; **C** Results of the sPLS-DA procedure trained on data of men

First, a pooled sPLS-DA analysis was performed to distinguish between successful and unsuccessful recanalization based on the BCC clusters, with an average classification error rate of 0.43 (SD = 0.02) (Fig. 1, Step 3 and 4). Sex-stratified analyses showed that the error rate was significantly lower for the female-only models (0.41; SD = 0.02) compared to the pooled (t(192.4) = 5.9, p < 0.001), and male-only models (0.47; SD = 0.03) (t(182.6) = -15.6, p < 0.001) (Fig. 1, Step 4, Fig 2). These results were visually confirmed in the sPLS-DA plot, where successful and unsuccessful recanalization groups were further apart for women than men (Fig. 3).

### Generic and sex specific blood cell characteristics related to recanalization success and acute ischemic stroke etiology

**Fig. 4:**
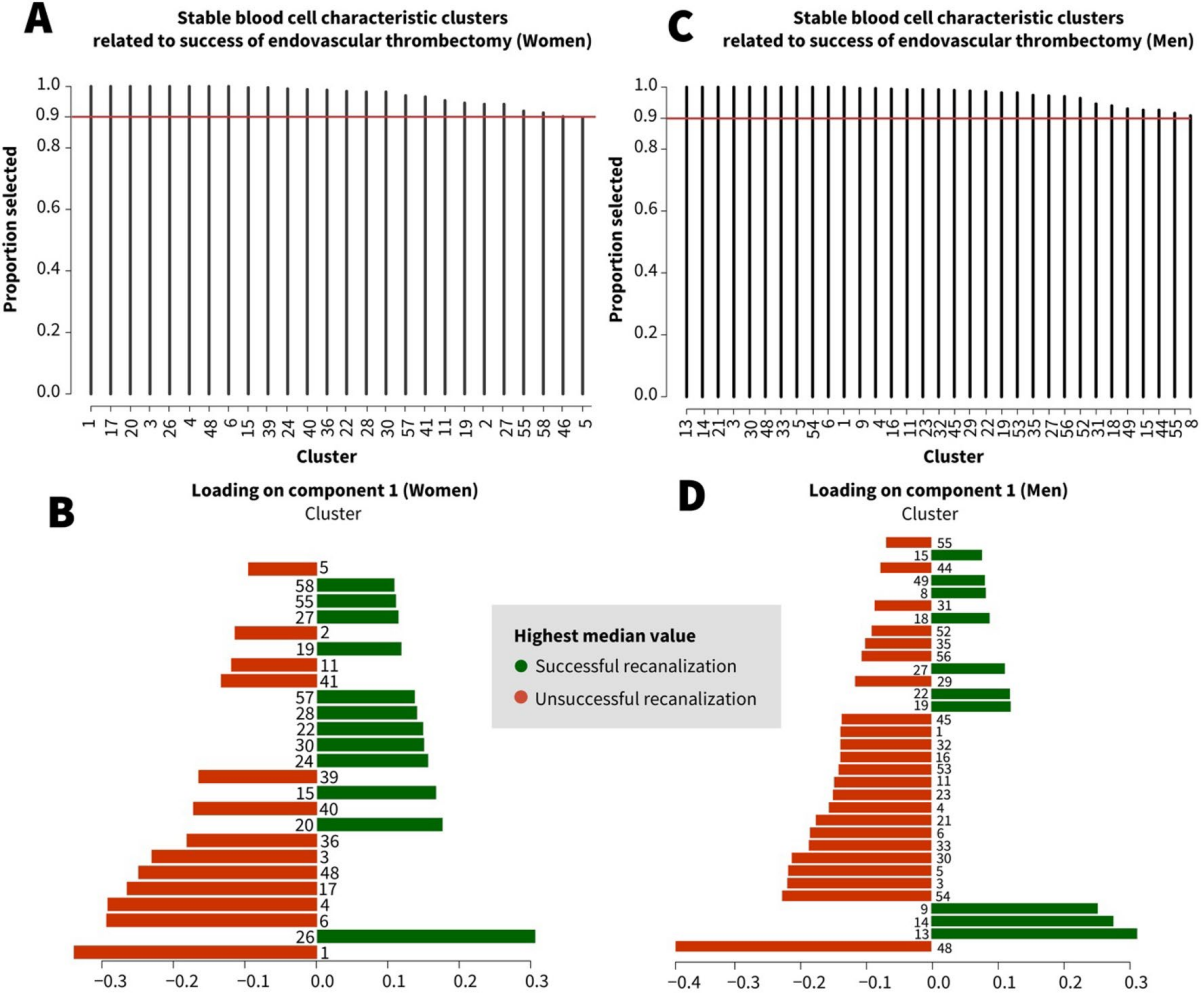
Selection frequency and loadings of BCC clusters in relation to recanalization success of endovascular thrombectomy in the female and male models across repeated cross-validation folds and repeats. **A**, **C** shows the frequency of selection across cross-validation folds and repeats for each BCC cluster for component 1. The stability threshold of 0.9 is indicated by the red line. Only BCCs with stability > 0.9 are shown. The loading plots **B**, **D** show the loadings of BCC clusters on the first sPLS-DA component, with the colors indicating in which EVT outcome group the median value was highest (green = successful, red = unsuccessful). For example, the reticulocyte percentage/absolute count (cluster 26) was selected in relation to recanalization success in 100% of the cross-validation folds and repeats in women (**A**), median reticulocyte percentage/absolute count (cluster 26) was higher in the successful recanalization group (**B**). Cluster 26 was not selected in relation to recanalization success in men

In women, 25 of the 58 BCC clusters were selected in relation to recanalization success in more than 90% across repeated cross-validation folds and repeats (Fig. 1, Step 5). In total, 13 of these clusters overlapped with men, 12 were female-specific (Fig. 4A, B). In men, 33 of the 58 BCC clusters fulfilled the prespecified criteria, of which 13 overlapped with women, 20 were male-specific (Fig. 4C, D).

**Fig. 5:**
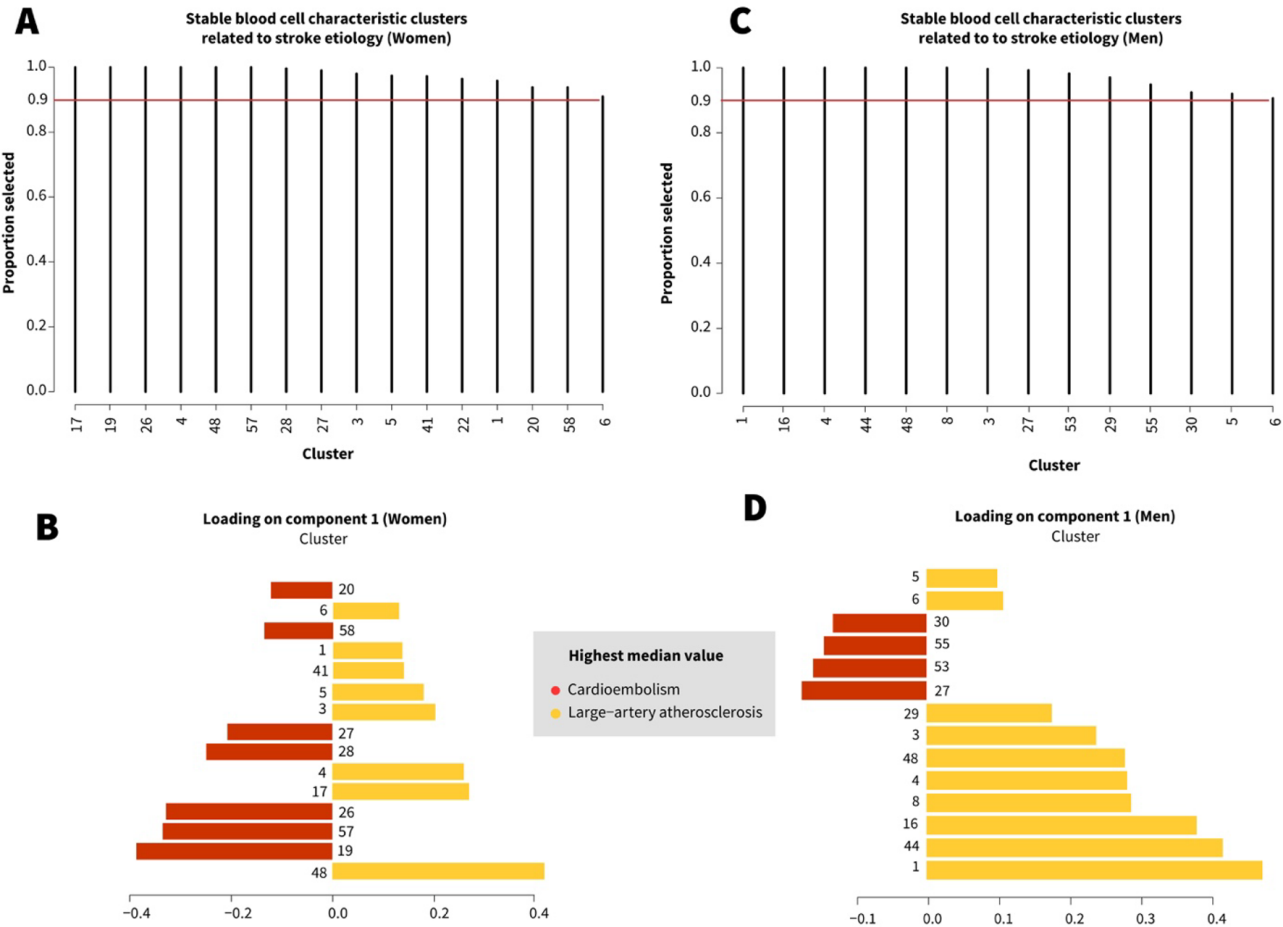
Selection frequency and loadings of BCC clusters previously related to recanalization success of endovascular thrombectomy (EVT), analyzed with  AIS etiology as outcome to provide biological meaning to the BCC clusters related to EVT success. **A**, **C** shows the frequency of selection across cross-validation folds and repeats for each BCC cluster for component 1. The stability threshold of 0.9 is indicated by the red line. Only BCCs with stability > 0.9 are shown. The loading plots **B**, **D** show the loadings of BCC clusters on the first sPLS-DA component, with the colors indicating in which AIS etiology group the median value was highest (red = cardioembolism, yellow = large-artery atherosclerosis). For example, hemoglobin/hematocrit (cluster 1) was selected in relation to recanalization success in 100% of the cross-validation folds and repeats in women (A), median hemoglobin/hematocrit (cluster 1) was higher in the unsuccessful recanalization group

In women, of the 25 stable BCC clusters related to recanalization success, 15 clusters were also being robust and informative for AIS etiology (Fig. 1, Step 6). In total, 3 of these overlapped with men, including the coefficient of variance of lymphocyte complexity of intracellular structure (cluster 48), and the absolute white blood cell count (cluster 4), with elevated levels in women and men with LAA as the cause of stroke, and the immature reticulocyte fraction (cluster 27), with elevated levels in women and men with a cardioembolism as the cause of stroke. The other 12 were female-specific (Fig. 5A, B), including primarily erythrocyte-related BCC clusters with elevated levels in patients with a cardioembolism as the cause of stroke, i.e. the red blood cell distribution width (cluster 19), reticulocyte count and reticulocyte percentage (cluster 26), and the percent of erythrocytes with hemoglobin concentration < 28 g/dL (cluster 28), among others, and leucocyte-related BCC clusters with elevated levels in LAA as the cause of stroke, i.e. the white blood cell viability fraction (cluster 5), the neutrophilic granulocyte absolute count (cluster 6), and neutrophil granularity (cluster 39), among others.

In men, of the 33 BCC clusters associated with recanalization success, 14 fulfilled the prespecified criteria for being robust and informative for AIS etiology. In total, 3 overlapped with women as mentioned above, 11 were male-specific (Fig. 5C, D), including primarily leucocyte-related BCCs with elevated levels in LAA as the cause of stroke, i.e. the monocyte absolute count (cluster 8), % basophilic granulocytes (cluster 16), and coefficient of variance of DNA/RNA staining in neutrophils (cluster 44), and erythrocyte-related BCCs with elevated levels in cardioembolism as the cause of strokey, i.e. hemoglobin distribution width (cluster 30), and red blood cell complexity of intracellular structure (cluster 53).

### Blood cell characteristics related to recanalization success and acute ischemic stroke etiology after controlling for cardiovascular risk factors

In women, after controlling for known cardiovascular risk factors, IVT prior to EVT (β = 0.37, 95% CI = 0.15–5.50, p = 0.026), and higher levels of the reticulocyte count and reticulocyte percentage (cluster 26) (β = 1.02, 95% CI = 0.03–1.51, p = 0.045), were associated with successful recanalization (Supplemental Table S3) (Fig. 1, Step 7). Higher levels of the mean corpuscular hemoglobin concentration (cluster 3) were associated with unsuccessful recanalization (β = − 0.55, 95% CI = -0.97 to − 0.02, p = 0.043). Thus, in combination with previous results, higher reticulocyte levels were associated with increased recanalization success and cardioembolism as the cause of stroke, and higher levels of the mean corpuscular hemoglobin concentration were associated with unsuccessful recanalization and LAA as the cause of stroke in women only.

In men, after controlling for cardiovascular risk factors, the coefficient of variance of lymphocyte complexity of intracellular structure (cluster 48) was associated with unsuccessful recanalization (β = − 0.60, 95% CI = − 0.97 to − 31, p = 0.001) (Supplemental Table S4). In combination with previous results, higher levels of the coefficient of variance of lymphocyte complexity of the intracellular structure was associated with unsuccessful recanalization and LAA as the cause of stroke in men only.

## Discussion

This study examined sex differences in blood cell characteristics (BCCs) associated with recanalization success and stroke etiology in patients with acute ischemic stroke (AIS) who underwent endovascular thrombectomy (EVT). Results showed that higher reticulocyte levels in women were linked to successful recanalization and cardioembolism as the cause of stroke, while elevated mean corpuscular hemoglobin concentration was associated with unsuccessful recanalization and LAA as the cause of stroke in women. In men, a higher coefficient of variance of lymphocyte complexity was associated with unsuccessful recanalization and LAA as the cause of stroke. These findings suggest sex-specific biological mechanisms underlying EVT recanalization success in AIS patients.

TICI 3 was achieved in around 50% of patients, similar to previous research [2, 24, 25]. with no difference between women and men. Prior research has shown conflicting results on sex differences in recanalization success. One study suggested that women have better outcomes [26], others have observed an unfavorable treatment effect for women [27], or no differences [10, 28]. The variability may be due to differences in study time frames, treatment efficacy, and the definition of successful recanalization[26].

### Potential biological mechanisms underlying EVT success in women and men

In our study, we found that 21 of 71 erythrocyte-, leucocyte- and platelet-related BCCs differed between sexes in the blood sample before EVT. Sex-specific reference intervals are commonly used to interpret hematology lab results due to known differences in BCCs between men and women [29–31]. This may be due to biological, hematological sex differences, as well as sex differences in response to AIS. Our results show that, in women, higher reticulocyte counts were associated with successful recanalization and cardioembolic stroke after correction for cardiovascular risk factors, which may have various causes and meanings, yet to be explored. Reticulocytes are immature erythrocytes that end up in the bloodstream during increased erythrocyte production in the bone marrow [32]. To speculate, erythrocyte-rich clot are associated with successful recanalization because of reduced clot friction and stiffness, and therefore have better integration of stents or conformity with an aspiration catheter, but erythrocyte-rich clots are also associated with distal migration or fragmentation of the clot [1, 4, 5, 7, 8, 33–35]. This may cause oxygen deprivation in the cerebral microvasculature, which potentially leads to increased production of red blood cells and the release of reticulocytes, immature red blood cells. Since women have lower hemoglobin levels, likely influenced by sex hormones [36], and therefore may have an earlier and stronger need for supplemental oxygen in case of vascular occlusion, this trigger of increased red blood cell production may be stronger than in men, providing a possible explanation for the relationship between elevated reticulocyte levels and recanalization success only in women.

Alternatively, atrial fibrillation can lead to (cardiac) hypoxia [37], which subsequently can lead to increased production of erythrocytes and reticulocytes in the bone marrow to increase oxygen supply to tissues. This may increase the risk of erythrocyte-rich clot formation through increased blood viscosity and higher plasma fibrin levels, related to the presence of an increased number of erythrocytes [38–41]. Women with atrial fibrillation have a higher risk of stroke than men [42, 43], possibly the lower hemoglobin level in women plays a role in this, so the trigger to produce extra erythrocytes which also releases reticulocytes is stronger in women than in men, but further research is needed to explore this.

In women, higher MCHC was linked to unsuccessful recanalization and LAA as the cause of stroke. MCHC measures the average hemoglobin in a single red blood cell and has been linked to carotid stenosis [44], coronary artery disease [45], and acute coronary syndrome [46]. Inflammation related to these conditions can cause oxidative stress, leading to hemolysis and increased MCHC [46]. Higher MCHC levels may indicate the presence of LAA in women, but the association with unsuccessful recanalization requires further investigation, in addition to the identified differences between men and women in this regard.

In men, a high coefficient of variance of lymphocyte complexity of the intracellular structure (LICV) is linked to unsuccessful recanalization and LAA as the cause of stroke. A high LICV suggests there are many lymphocytes with different intracellular structures, indicating the presence of different types of lymphocytes. Certain subsets of T lymphocytes, particularly T_H_1 cells, are known to drive and modify atherosclerosis, but the role of other T lymphocyte subsets is disputed [47]. Further research is needed to understand the meaning of increased LICV and why it is only associated with recanalization succes and LAA as cause of stroke in men and not in women, since LICV is a novel, unexplored hematological marker.

### Clinical worth and implications

The results of this study have several implications. The blood cell characteristics used in this study, such as reticulocyte levels, MCHC, and LICV, could potentially serve as predictors of EVT success and AIS etiology in women and men. The ease of measuring these markers in an emergency setting makes them promising candidates for implementation in daily clinical care. Incorporating these blood cell characteristics into the diagnostic process might aid clinicians in selecting appropriate treatments and interventions for stroke patients, leading to more personalized and effective care. However, these potentially novel biomarkers should be interpreted in the clinical reasoning process, which requires additional prospective cohort studies to further explore their significance. However, as the error rates of the sex-stratified sPLS-DA models are still relatively high, it should be realized that the blood cell characteristics do not provide 100% certainty about the success of EVT and AIS etiology. Adding additional clinical variables to the model could improve the model performance, to make them potentially supportive in the clinical reasoning process when the results are externally validated. In addition, to understand the exact significance of blood cell characteristics in stroke pathophysiology, the specific markers must be further investigated. Because of the study design, it cannot be determined whether blood cell characteristics derived from circulating blood are involved in thrombus formation, stroke response, or other factors, to determine whether they also have therapeutic implications. Histological data from the thrombus, for example, could be added to further investigate relationships between and thrombus morphology and the blood cell characteristics. Also, a link between targeted proteomic panels and blood cell characteristics could also be established in a similar study setting to understand biological processes at the protein level.

This study has several strengths, including the use of machine learning to gain insight into sex differences in BCCs related to AIS, which is novel. Additionally, selection bias was low because all BCCs were automatically measured and extracted when a hemoglobin measurement was requested. Lastly, routine care data were used in cohort selection, making the results consistent with and generalizable to clinical practice.

This study has limitations. Still, a form of selection bias was present as patients with no available BCCs within 60 min after presentation were excluded, and the study was conducted in a tertiary referral center where many patients were referred for EVT only and had blood drawn elsewhere. Missing data was also a challenge, as stroke-to-door and door-to-needle times were not always stored in the stroke questionnaires, and relevant information about the group of referred patients from another center is missing. Choosing a missing data imputation strategy in this context is complicated, which may have influenced results related to vascular risk factors. Further research is needed to address these questions.

## Conclusions

With extensive machine learning analyses on routine care data from patients with acute ischemic stroke, we identified sex differences in blood cell characteristics associated with the success of endovascular thrombectomy and the etiology of the stroke. This study highlights the potential of analyzing data from routine care flow cytometry analyzers to identify sex-differences in factors related to acute ischemic stroke, which may ultimately facilitate personalized treatment.

### Supplementary Information

Below is the link to the electronic supplementary material.

Supplementary file1 (DOCX 407 kb)

## Data Availability

Please contact the authors for data requests.
