# Supplementary material for: Sexual dimorphism in peripheral blood cell characteristics linked to recanalization success of endovascular thrombectomy in acute ischemic stroke
Source: J Thromb Thrombolysis. 2023 Aug 18;56(4):614–25. doi: 10.1007/s11239-023-02881-z (PMC10550865; doi:10.1007/s11239-023-02881-z)
Supplement: Supplementary file 1 — Supplementary file1 (DOCX 407 kb) [file 11239_2023_2881_MOESM1_ESM.docx]

**Supplemental Methods Tables, and Figures**

**Supplemental Methods**

*Step-by-step analysis*

**Step 1: Comparing blood cell characteristics between women and men**

First, all BBCs were compared between women and men irrespective of their relationship with recanalization success, given known biological sex differences in some of these characteristics, like hemoglobin levels and the absolute platelet count[[30], [31]](https://sciwheel.com/work/citation?ids=14337636,9082325&pre=&pre=&suf=&suf=&sa=0,0&dbf=0&dbf=0) using Wilcoxon rank sum tests with a False discovery rate correction for multiple testing.

**Step 2: Clustering blood cell characteristics**

The BCCs contain multicollinearity as for example hemoglobin and hematocrit levels are closely correlated. Multicollinearity can obscure the true impact of each variable on the discriminant function, leading to difficulties in interpreting and explaining the results, and should therefore be addressed. Using the ClustOfVar R package[[48]](https://sciwheel.com/work/citation?ids=14337776&pre=&suf=&sa=0&dbf=0), we identified clusters of BCCs within which the characteristics are strongly related. Synthetic variables derived from the first principal component that represented the BCCs in the clusters were created, to eliminate multicollinearity. The appropriate number of clusters was determined with a bootstrap approach, i.e., the stability function from the ClustOfVar package[[48]](https://sciwheel.com/work/citation?ids=14337776&pre=&suf=&sa=0&dbf=0). All synthetic variables were mean-centered, and these variables were used in the following analyses. We performed this analysis in a sex-stratified manner.

**Step 3: Performing Partial Least Squares Discriminant Analysis (sPLS-DA)**

The analysis to identify BCC clusters related to recanalization success in women and men was largely based on the machine learning technique sparse Partial Least Squares Discriminant Analysis (sPLS-DA), using the mixOmics R package[[49]](https://sciwheel.com/work/citation?ids=1510331&pre=&suf=&sa=0&dbf=0). sPLS-DA creates components based on the BCC clusters that best distinguish the two recanalization success groups. sPLS-DA is a method to extract biologically relevant information in high-dimensional datasets[[49]](https://sciwheel.com/work/citation?ids=1510331&pre=&suf=&sa=0&dbf=0). Furthermore, sPLS-DA performs variable selection by including a Lasso penalty on the loading vectors and can select an optimal number of components to use from the perspective of parsimony.

**Step 4: Assessing the added value of sex stratifying blood cell characteristic clusters in distinguishing recanalization success**

Next, to assess the added value of sex-stratified analyses to distinguish successful from unsuccessful recanalization, three sPLS-DA models were trained, one with pooled data of women and men to use as a point of reference, and two with data of women and men separately. Repeated 5-fold cross-validation was applied 100 times. To test whether sex-stratified analyses led to better discriminability of the models, we analyzed the differences in error rate distributions of the three models across the cross-validation folds and replications using Welch two sample t-tests. The error rate was defined as the number of misclassified samples divided by the total number of samples.

**Step 5: Identifying stable blood cell characteristic clusters related to recanalization success in women and men separately**

Then, to determine which BCC clusters were related to recanalization success in women and men, a stability selection analysis was performed to assess the robustness of the BCC clusters in relation to recanalization success over the repeated cross-validation folds[[50]](https://sciwheel.com/work/citation?ids=1510296&pre=&suf=&sa=0&dbf=0). BCC clusters that were selected in more than 90% of the selections across repeated cross-validation folds, which is an arbitrary but common cutoff [[50]](https://sciwheel.com/work/citation?ids=1510296&pre=&suf=&sa=0&dbf=0), were considered informative and stable for distinguishing in relation to recanalization success.

**Step 6: Identifying stable blood cell characteristic clusters related to acute ischemic stroke etiology in women and men separately**

To explore potential biological meaning to the identified BCCs related to recanalization success, we performed the sPLS-DA procedure on the subsets of BCCs found to be related to recanalization success in women and men and repeated the above sPLS-DA-procedure with acute ischemic stroke etiology (cardioembolic, large-artery atherosclerosis) as outcome. Again, the stability of the selected BCCs over the repeated 5-fold cross-validation procedure was assessed and characteristics that were selected in more than 90% of the selections across repeated cross-validation folds were considered informative in relation to acute ischemic stroke etiology.

**Step 7: Determining the added value of BCCs in relation to recanalization success and acute ischemic stroke etiology after controlling for cardiovascular risk factors**

Subsequently, to explore additional potential biological meaning, BCCs that were considered both stable in relation to recanalization success and acute ischemic stroke etiology were again tested in relation to recanalization success, now considering known cardiovascular risk factors. This was done using generalized logistic regression with the glmnet package in R[[51]](https://sciwheel.com/work/citation?ids=171561&pre=&suf=&sa=0&dbf=0), by including a penalty value λ on the model coefficients to reduce overfitting. The optimal penalty value λ was chosen using tenfold cross-validation [[51]](https://sciwheel.com/work/citation?ids=171561&pre=&suf=&sa=0&dbf=0). Well-known cardiovascular risk factors and BCCs found to be related to recanalization success and acute ischemic stroke etiology were included in the models. This method leaves a subset of variables with a generalized coefficient explaining the relationship between the BCC and recanalization success considering the other covariates, if no effect is found the coefficient is reduced to 0. P-values and confidence intervals for the lasso estimate were calculated using the fixedLassoInf function from the selectiveInference R package[[52]](https://sciwheel.com/work/citation?ids=14337620&pre=&suf=&sa=0&dbf=0).

**Supplemental Tables**

**Supplemental Table S1.** Blood cell characteristics (BCCs), their abbreviations, and the cluster they belong to. The cluster-classification was determined with a bootstrap approach, i.e. the stability function from the ClustOfVar R package. Based on this classification, synthetic variables based on the first principal component were created to eliminate multicollinearity. Table fields are colored according to the cluster-classification, for reading convenience.

| Abbreviation | BCC | Unit | Cluster |
| --- | --- | --- | --- |
| HB | Hemoglobin SI-units | mmol/L | 1 |
| HGB | Hemoglobin USA-units | g/dL | 1 |
| HT | Hematocrit | Fraction | 1 |
| MCH | Mean Corpuscular Hemoglobin (MCH) | fmol | 2 |
| MCH USA | Mean Corpuscular Hemoglobin (MCH) USA units | AU | 2 |
| MCHC | Mean Corpuscular Hemoglobin Concentration (MCHC) | mmol/L | 3 |
| MCHC USA | Mean Corpuscular Hemoglobin Concentration (MCHC) USA units | g/dL | 3 |
| WBC | White Blood Cell Count | 10^9/L | 4 |
| WVF | White blood cell viability fraction | Fraction | 5 |
| NEU | Neutrophilic granulocyte absolute count | 10^9/L | 6 |
| SEG | Segmented granulocyte absolute count | 10^9/L | 6 |
| LYM | Lymphocyte absolute count | 10^9/L | 7 |
| LYME | Lymphocyte absolute count (excluding atypical lymphocytes) | 10^9/L | 7 |
| MON | Monocyte absolute count | 10^9/L | 8 |
| MONE | Monocytes (excluding blasts) absolute count | 10^9/L | 8 |
| EOS | Eosinophil granulocyte absolute count | 10^9/L | 9 |
| BAS | Basophilic granulocyte absolute count | 10^9/L | 10 |
| PNEU | Percentage neutrophilic granulocytes | % | 11 |
| PSEG | Segmented granulocyte percentage count | % | 11 |
| PLYM | Percentage of lymphocytes | % | 12 |
| PLYME | Percentage of lymphocytes (excluding atypical lym) | % | 12 |
| PMON | Percentage of monocytes | % | 13 |
| PMONE | Percentage of monocytes (excluding blasts) | % | 14 |
| PEOS | Percentage of eosinophilic granulocytes | % | 15 |
| PBAS | Percentage of basophilic granulocytes | % | 16 |
| RBCI | Red Blood Cell (RBC) Count by Impedance (RBCi) | 10^12/L | 17 |
| RBCO | Red Blood Cell (RBC) Count by Optics (RBCo) | 10^12/L | 17 |
| MCV | Mean Corpuscular Volume (MCV) | fL | 18 |
| RDW | RBC Distribution Width | % CV | 19 |
| PMIC | Percentage of RBCs with volume less than 60 fL | % | 20 |
| PMAC | Percentage of RBCs with volume greater than 120 fL | % | 21 |
| PLT | Platelet count | 10^9/L | 22 |
| PLTI | Platelet Count by Impedance | 10^9/L | 22 |
| PLTO | Platelet Count by Optics | 10^9/L | 22 |
| MPV | Mean Platelet Volume (MPV) | fL | 23 |
| PCT | Plateletcrit (PCT) | % | 24 |
| PDW | Platelet Distribution Width (PDW) | AU | 25 |
| PRETC | Percentage of reticulocytes | % | 26 |
| RETC | Reticulocyte absolute count | 10^9/L | 26 |
| IRF | Immature Reticulocyte Fraction | Fraction | 27 |
| PHPO | Percent of RBCs with HGB concentration less than 28 g/dL | % | 28 |
| PHPR | Percent of RBCs with HGB concentration more than 41 g/dL | % | 29 |
| HDW | Hemoglobin Distribution Width | AU | 30 |
| MCVR | Mean Corpuscular Volume of Reticulocytes (MCVr) | AU | 31 |
| MCHR | Mean Corpuscular HGB per Reticulocyte (MCHr) | fmol | 32 |
| MCHCR | Mean Corpuscular HGB Concentration per Reticulocyte (MCHCr) | mmol/L | 33 |
| PRP | Percentage of reticulated platelets / Enumeration of reticulated platelets | % | 34 |
| NAMN | Axial Light Loss; Mean neutrophil size | AU | 35 |
| NACV | Coefficient of variance of neutrophil size | CV | 36 |
| NIMN | Intermediate Angle Scattering; Neutrophil complexity of intracellular structure | AU | 37 |
| NICV | Coefficient of variance of neutrophil complexity of intracellular structure | CV | 38 |
| NPMN | Neutrophil Lobularity/Granularity and nuclear segmentation | AU | 39 |
| NPCV | Coefficient of variance of Neutrophil Lobularity/Granularity and nuclear segmentation | CV | 40 |
| NDMN | Neutrophil Lobularity/Granularity and nuclear lobularity | AU | 41 |
| NDCV | Coefficient of variance of Neutrophil depolarization | CV | 42 |
| NFMN | DNA/RNA staining in neutrophils (viability) | AU | 43 |
| NFCV | Coefficient of variance of DNA/RNA staining in neutrophils (viability) | CV | 44 |
| LAMN | Axial Light Loss; Mean lymphocyte size | AU | 45 |
| LACV | Coefficient of variance of lymphocyte size | CV | 46 |
| LIMN | Lymphocyte complexity of intracellular structure | AU | 47 |
| LICV | Coefficient of variance of lymphocyte complexity of intracellular structure | CV | 48 |
| PIMN | Intermediate Angle Scattering; platelet complexity of intracellular structure | AU | 49 |
| PICV | Coefficient of variance of platelet complexity of intracellular structure | CV | 50 |
| PPMN | Polarized Side Scattering; platelet Lobularity | AU | 51 |
| PPCV | Coefficient of variance of platelet Lobularity | CV | 52 |
| RBCIMN | Intermediate Angle Scattering; red blood cell complexity of intracellular structure | AU | 53 |
| RBCICV | Coefficient of variance of red blood cell complexity of intracellular structure | CV | 54 |
| RBCFMN | Mean of FL1 signal - larger than 0: presence of NRBCs | AU | 55 |
| RBCFCV | CV% of FL1 signal - larger than 0: presence of NRBCs | CV | 56 |
| RTCFMN | Position of Reticulocyte population during reticulocyte measurement on FL1 signal - reticulated platelets | AU | 57 |
| RTCFCV | CV% of Reticulocyte population during reticulocyte measurement on FL1 signal - reticulated platelets | CV | 58 |

**Supplemental Table S2.** Blood cell characteristics (BCCs) at baseline were compared between women and men. A false discovery rate correction for multiple testing was applied.

| Variable | N | F, N = 143*^1^* | M, N = 190*^1^* | p-value*^2^* | q-value*^3^* |
| --- | --- | --- | --- | --- | --- |
| PLTI | 333 | 262.9 (231.1, 318.9) | 210.7 (173.7, 259.0) | <0.001 | <0.001 |
| PCT | 333 | 0.2 (0.2, 0.2) | 0.2 (0.1, 0.2) | <0.001 | <0.001 |
| PLT | 333 | 269.0 (228.5, 325.5) | 217.2 (178.0, 267.0) | <0.001 | <0.001 |
| PLTO | 333 | 269.0 (228.5, 325.5) | 217.2 (178.0, 267.0) | <0.001 | <0.001 |
| RBCICV | 333 | 1.5 (1.5, 1.7) | 1.6 (1.5, 1.8) | <0.001 | <0.001 |
| HB | 333 | 8.5 (7.9, 9.1) | 9.0 (8.3, 9.6) | <0.001 | <0.001 |
| HB (USA) | 333 | 13.7 (12.8, 14.7) | 14.6 (13.4, 15.5) | <0.001 | <0.001 |
| PHPR | 333 | 0.0 (0.0, 0.0) | 0.0 (0.0, 0.1) | <0.001 | <0.001 |
| PMONE | 333 | 6.6 (5.2, 8.6) | 7.6 (6.4, 9.7) | <0.001 | <0.001 |
| MCHR | 333 | 29.9 (28.4, 31.2) | 30.9 (29.5, 32.0) | <0.001 | <0.001 |
| PMON | 333 | 6.6 (5.2, 8.6) | 7.6 (6.4, 9.7) | <0.001 | <0.001 |
| HT | 333 | 41.2 (38.2, 43.3) | 42.9 (39.5, 45.5) | <0.001 | 0.002 |
| PICV | 333 | 16.7 (16.2, 17.2) | 16.9 (16.4, 17.7) | 0.003 | 0.018 |
| MCHCR | 333 | 29.6 (28.5, 30.4) | 29.9 (29.1, 31.0) | 0.004 | 0.022 |
| PHPO | 333 | 2.6 (1.1, 5.3) | 1.5 (0.7, 3.9) | 0.005 | 0.023 |
| MCH | 333 | 1.9 (1.8, 2.0) | 1.9 (1.9, 2.0) | 0.006 | 0.023 |
| MCH (USA) | 333 | 30.5 (29.5, 31.9) | 31.1 (30.0, 32.1) | 0.006 | 0.023 |
| RBCO | 333 | 4.5 (4.2, 4.8) | 4.7 (4.3, 5.0) | 0.006 | 0.023 |
| PMIC | 333 | 0.7 (0.5, 1.0) | 0.6 (0.5, 0.8) | 0.007 | 0.027 |
| RBCI | 333 | 4.5 (4.2, 4.8) | 4.7 (4.3, 5.0) | 0.010 | 0.034 |
| PPCV | 333 | 12.6 (12.1, 13.2) | 12.9 (12.2, 13.7) | 0.010 | 0.034 |
| PDW | 333 | 16.1 (15.7, 16.5) | 16.2 (15.8, 16.7) | 0.016 | 0.050 |
| LIMN | 333 | 78.0 (76.3, 79.8) | 77.3 (74.8, 79.2) | 0.016 | 0.050 |
| MCVR | 333 | 100.1 (97.0, 103.1) | 101.4 (97.8, 105.6) | 0.029 | 0.086 |
| MCHC | 333 | 0.2 (0.2, 0.2) | 0.2 (0.2, 0.2) | 0.039 | 0.10 |
| MCHC (USA) | 333 | 33.6 (32.9, 34.4) | 33.9 (33.2, 34.5) | 0.039 | 0.10 |
| PRETC | 333 | 1.4 (1.2, 1.8) | 1.4 (1.1, 1.7) | 0.040 | 0.10 |
| MONE | 333 | 0.7 (0.5, 0.9) | 0.7 (0.6, 0.9) | 0.040 | 0.10 |
| NDCV | 333 | 15.4 (14.4, 16.3) | 15.0 (14.2, 15.8) | 0.043 | 0.10 |
| HDW | 333 | 7.0 (6.5, 7.7) | 7.2 (6.7, 7.8) | 0.056 | 0.13 |
| MON | 333 | 0.7 (0.5, 0.9) | 0.7 (0.6, 0.9) | 0.063 | 0.14 |
| MCV | 333 | 91.0 (87.9, 93.3) | 91.6 (88.8, 94.8) | 0.066 | 0.15 |
| NFCV | 333 | 8.5 (7.7, 9.3) | 8.1 (7.2, 9.2) | 0.084 | 0.2 |
| NPCV | 333 | 8.4 (7.4, 9.6) | 8.1 (7.0, 9.2) | 0.11 | 0.2 |
| PIMN | 333 | 145.8 (141.1, 149.2) | 146.8 (142.9, 151.4) | 0.11 | 0.2 |
| NEU | 333 | 7.2 (5.1, 9.6) | 6.5 (4.5, 9.2) | 0.12 | 0.2 |
| SEG | 333 | 7.2 (5.1, 9.6) | 6.5 (4.5, 9.0) | 0.12 | 0.2 |
| PMAC | 333 | 1.7 (1.0, 2.6) | 1.8 (1.1, 3.2) | 0.14 | 0.3 |
| PPMN | 333 | 126.3 (122.8, 129.4) | 127.1 (123.3, 130.3) | 0.14 | 0.3 |
| PBAS | 333 | 0.4 (0.2, 0.6) | 0.5 (0.2, 0.7) | 0.2 | 0.3 |
| LAMN | 333 | 101.5 (98.6, 103.8) | 101.7 (99.5, 104.5) | 0.2 | 0.3 |
| PEOS | 333 | 1.2 (0.6, 2.3) | 1.3 (0.6, 2.7) | 0.2 | 0.3 |
| RTCFCV | 333 | 11.0 (10.4, 11.9) | 11.2 (10.6, 11.9) | 0.2 | 0.3 |
| WBC | 333 | 10.0 (7.6, 12.8) | 9.2 (7.5, 11.8) | 0.2 | 0.3 |
| RETC | 333 | 67.2 (51.5, 80.2) | 64.0 (51.5, 77.3) | 0.2 | 0.4 |
| WVF | 333 | 1.0 (1.0, 1.0) | 1.0 (1.0, 1.0) | 0.2 | 0.4 |
| PNEU | 333 | 72.8 (61.9, 81.7) | 70.4 (61.5, 78.7) | 0.3 | 0.4 |
| EOS | 333 | 0.1 (0.1, 0.2) | 0.1 (0.1, 0.2) | 0.3 | 0.5 |
| PSEG | 333 | 72.7 (61.9, 80.3) | 70.1 (61.5, 78.4) | 0.4 | 0.5 |
| LACV | 333 | 4.4 (3.7, 5.7) | 4.4 (3.4, 5.4) | 0.4 | 0.6 |
| RBCFCV | 333 | 11.2 (9.5, 12.8) | 11.3 (9.7, 13.2) | 0.5 | 0.7 |
| LICV | 333 | 4.4 (3.8, 5.1) | 4.6 (3.8, 5.1) | 0.5 | 0.7 |
| NICV | 333 | 3.7 (3.3, 4.0) | 3.7 (3.4, 4.0) | 0.5 | 0.7 |
| BAS | 333 | 0.0 (0.0, 0.1) | 0.0 (0.0, 0.1) | 0.5 | 0.7 |
| NPMN | 333 | 126.5 (120.1, 136.5) | 128.5 (120.1, 135.3) | 0.6 | 0.7 |
| NFMN | 333 | 70.8 (69.0, 71.7) | 70.4 (68.7, 71.8) | 0.6 | 0.7 |
| RBCIMN | 333 | 182.0 (180.3, 184.9) | 182.0 (179.8, 184.9) | 0.6 | 0.8 |
| MPV | 333 | 7.4 (6.8, 8.1) | 7.5 (6.9, 8.1) | 0.6 | 0.8 |
| RTCFMN | 333 | 133.3 (130.5, 136.6) | 133.6 (130.8, 136.3) | 0.6 | 0.8 |
| NDMN | 333 | 28.5 (26.4, 30.6) | 28.0 (25.9, 30.9) | 0.7 | 0.9 |
| PRP | 333 | 2.7 (2.0, 3.9) | 2.8 (2.0, 3.9) | 0.8 | >0.9 |
| RBCFMN | 333 | 85.2 (82.5, 87.6) | 85.0 (82.2, 87.5) | 0.8 | >0.9 |
| LYM | 333 | 1.7 (1.1, 2.3) | 1.6 (1.2, 2.3) | 0.8 | >0.9 |
| LYME | 333 | 1.7 (1.1, 2.3) | 1.6 (1.2, 2.3) | 0.8 | >0.9 |
| PLYM | 333 | 18.1 (10.4, 27.6) | 18.2 (12.3, 25.7) | 0.9 | >0.9 |
| PLYME | 333 | 18.1 (10.4, 27.6) | 18.2 (12.3, 25.7) | 0.9 | >0.9 |
| NAMN | 333 | 143.1 (137.8, 150.0) | 143.3 (138.5, 148.0) | 0.9 | >0.9 |
| NACV | 333 | 2.6 (2.4, 2.9) | 2.6 (2.4, 2.9) | >0.9 | >0.9 |
| RDW | 333 | 12.2 (11.5, 12.9) | 12.1 (11.7, 12.8) | >0.9 | >0.9 |
| NIMN | 333 | 140.1 (136.4, 144.1) | 140.5 (135.4, 144.3) | >0.9 | >0.9 |
| IRF | 333 | 0.3 (0.2, 0.3) | 0.3 (0.2, 0.3) | >0.9 | >0.9 |
| *^1^* Median (IQR) | | | | | |
| *^2^* Wilcoxon rank sum test | | | | | |
| *^3^* False discovery rate correction for multiple testing | | | | | |

**Supplemental Table S3.** Results of a generalized logistic regression model trained on data of females with blood cell characteristic clusters previously related to recanalization success, controlling for known cardiovascular risk factors.

| Variable | β | Z-score | P-value | Low conf pt | Up conf pt |
| --- | --- | --- | --- | --- | --- |
| Age | -0.398 | -1.917 | 0.056 | -0.740 | 0.015 |
| Intravenous thrombolysis prior to MT | 0.370 | 1.647 | **0.026*** | 0.149 | 5.503 |
| Hypertension | 0 | - | - | - | - |
| Hyperlipidemia | 0 | - | - | - | - |
| Atrial fibrillation | 0 | - | - | - | - |
| Diabetes Mellitus | 0 | - | - | - | - |
| Current smoker | 0 | - | - | - | - |
| History of stroke or transient ischemic attack | 0 | - | - | - | - |
| History of myocardial infarction | 0 | - | - | - | - |
| Platelet aggregation inhibitor use | 0 | - | - | - | - |
| Direct oral anticoagulant use | 0 | - | - | - | - |
| Cluster 48 (CV of lymphocyte complexity of intracellular structure) | -0.270 | -1.323 | 0.591 | -0.528 | 1.428 |
| Cluster 19 (RBC distribution width) | -0.400 | -0.911 | 0.357 | -1.367 | 1.143 |
| Cluster 57 (Reticulated platelets (mean)) | 0.870 | 2.352 | 0.051 | -0.011 | 1.476 |
| Cluster 26 (Reticulocyte count and reticulocyte %) | 1.019 | 3.324 | **0.045*** | 0.034 | 1.511 |
| Cluster 17 (RBC count) | -1.103 | -1.869 | 0.170 | -2.054 | 0.861 |
| Cluster 4 (WBC count) | 0.041 | 0.068 | 0.955 | -32.395 | -0.080 |
| Cluster 28 (% RBCs with HGB < 28 g/dL) | -0.296 | -0.851 | 0.620 | -0.764 | 2.597 |
| Cluster 27 (Immature reticulocyte fraction) | -1.834 | -2.892 | 0.289 | -2.711 | 2.789 |
| Cluster 3 (Mean corpuscular hemoglobin concentration) | -0.547 | -2.135 | **0.043*** | -0.970 | -0.024 |
| Cluster 5 (White blood cell viability fraction) | 0.642 | 2.688 | 0.053 | 0.207 | 1.035 |
| Cluster 41 (Neutrophil lobularity) | -0.408 | -1.959 | 0.051 | -0.754 | 0.002 |
| Cluster 1 (Hemoglobin and hematocrit) | 0.763 | 1.186 | 0.240 | -1.043 | 1.817 |
| Cluster 58 (Reticulated platelets (CV)) | 1.001 | 2.596 | 0.238 | -1.185 | 1.570 |
| Cluster 6 (Neutrophilic granulocyte and segmented granulocyte count) | -0.977 | -1.479 | 0.890 | -0.923 | 31.268 |
| Cluster 20 (RBC with volume < 60 fL) | 2.818 | 2.125 | 0.066 | -0.311 | 4.993 |

The model includes a penalty value λ on the model coefficients to reduce overfitting. This method leaves a subset of variables with a generalized coefficient explaining the relationship between BCC and MT efficacy considering known cardiovascular risk factors, if no effect is found the coefficient is reduced to 0. P-values and confidence intervals for the lasso estimate were calculated using the fixedLassoInf function from the selectiveInference R package..

**Supplemental Table S4.** Results of a generalized logistic regression model trained on data of females with blood cell characteristic clusters previously related to recanalization success, controlling for known cardiovascular risk factors.

| Variable | β | Z-score | P-value | Low conf pt | Up conf pt |
| --- | --- | --- | --- | --- | --- |
| Age | 0.217 | 1.289 | 0.115 | -0.170 | 1.649 |
| Intravenous thrombolysis prior to MT | -0.033 | -0.192 | 0.705 | -0.954 | 2.746 |
| Hypertension | 0 | - | - | - | - |
| Hyperlipidemia | 0 | - | - | - | - |
| Atrial fibrillation | 0 | - | - | - | - |
| Diabetes Mellitus | 0 | - | - | - | - |
| Current smoker | 0 | - | - | - | - |
| History of stroke or transient ischemic attack | 0 | - | - | - | - |
| History of myocardial infarction | 0 | - | - | - | - |
| Platelet aggregation inhibitor use | 0 | - | - | - | - |
| Direct oral anticoagulant use | 0 | - | - | - | - |
| Cluster 1 (Hemoglobin and hematocrit) | 0.244 | 0.684 | 0.267 | -1.253 | 3.549 |
| Cluster 44 (DNA/RNA staining in neutrophils) | 0.193 | 1.247 | 0.261 | -0.298 | 0.443 |
| Cluster 16 (Basophilic granulocyte %) | -0.263 | -0.735 | 0.243 | -3.680 | 1.122 |
| Cluster 8 (Monocyte absolute count) | 0.296 | 1.296 | 0.807 | -5.466 | 0.566 |
| Cluster 4 (White blood cell count) | -0.045 | -0.072 | 0.904 | -0.881 | 22.761 |
| Cluster 48 (CV of lymphocyte complexity of intracellular structure) | -0.599 | -3.718 | **0.001**** | -0.972 | -0.308 |
| Cluster 3 (Mean corpuscular hemoglobin concentration) | -0.202 | -1.067 | 0.203 | -1.436 | 0.384 |
| Cluster 29 (% RBCs with HGB > 41 g/dL) | -0.035 | -0.215 | 0.653 | -1.032 | 2.219 |
| Cluster 27 (Immature reticulocyte fraction) | 0.208 | 1.237 | 0.438 | -0.722 | 0.489 |
| Cluster 53 (RBC complexity of intracellular structure) | -0.932 | -1.027 | 0.307 | -5.470 | 2.879 |
| Cluster 55 (Presence of nucleated red blood cells) | 0.038 | 0.109 | 0.909 | -9.531 | 0.277 |
| Cluster 30 (Hemoglobin distribution width) | -0.279 | -1.369 | 0.143 | -1.113 | 0.210 |
| Cluster 6 (neutrophilic granulocyte and segmented granulocyte count) | -0.478 | -0.851 | 0.092 | -19.698 | 0.876 |
| Cluster 5 (White blood cell viability fraction) | -0.077 | -0.468 | 0.639 | -0.312 | 0.989 |

The model includes a penalty value λ on the model coefficients to reduce overfitting. This method leaves a subset of variables with a generalized coefficient explaining the relationship between BCC and recanalization success considering known cardiovascular risk factors, if no effect is found the coefficient is reduced to 0. P-values and confidence intervals for the lasso estimate were calculated using the fixedLassoInf function from the selectiveInference R package (24).

**Supplemental Figures**

**Supplemental Figure S1.** Sapphire Blood cell characteristics (BCCs) and clusters they belong to are shown. The number of clusters (N=58 was determined with a bootstrap approach, i.e. the stability function from the ClustOfVar R package. With 58 clusters, the highest Rand-index was achieved (0.98), indicating that this was the most stable cluster-classification across all bootstraps. Based on this clustering, synthetic variables were created to eliminate multicollinearity. BCCs with the same color belong to the same cluster.
